# Rare versus common diseases: a false dichotomy in precision medicine

**DOI:** 10.1038/s41525-021-00176-x

**Published:** 2021-02-24

**Authors:** Brian Hon Yin Chung, Jeffrey Fong Ting Chau, Gane Ka-Shu Wong

**Affiliations:** 1 Department of Paediatrics & Adolescent Medicine, Li Ka Shing Faculty of Medicine, The University of Hong Kong, Hong Kong, China; 2 Department of Medicine and Department of Biological Sciences, The University of Alberta, Edmonton, AB Canada; 3 BGI-Shenzhen, Beishan Industrial Zone, Yantian District, Shenzhen, China

**Keywords:** Genetic testing, Drug development

## Abstract

Precision medicine initiatives are being launched worldwide, each with the capacity to sequence many thousands to millions of human genomes. At the strategic planning level, all are debating the extent to which these resources will be directed towards rare diseases (and cancers) versus common diseases. However, these are not mutually exclusive choices. The organizational and governmental infrastructure created for rare diseases is extensible to common diseases. As we will explain, the underlying technology can also be used to identify drug targets for common diseases with a strategy focused on naturally occurring human knockouts. This flips on its head the prevailing modus operandi of studying people with diseases of interest, shifting the onus to defining traits worth emulating by pharmaceuticals, and searching phenotypically for people with these traits. This also shifts the question of what is rare or common from the many underlying causes to the possibility of a common final pathway.

## Introduction

The 100,000 Genomes Project led by Genomics England has been a huge success, based not only on their scientific publications^1^ but also by their impact on the National Health Service (NHS). Since 2019, NHS has offered genome sequencing as part of healthcare, and the plan is to sequence five million individuals over the next 5 years^2^. This has inspired similar initiatives worldwide, even in middle-income countries like Thailand^3^. Many are focusing on rare diseases, or to a lesser extent cancers. Others are studying the general population and/or building infrastructure (see Table 1). This reflects a longstanding categorization of medical disorders as rare diseases of primarily monogenic etiology versus common diseases of complex multifactorial etiology where most of the healthcare spending resides. These projects all envision a future of precision medicine (PM) where the availability of more data (not necessarily always genomes) facilitates our ability to better diagnose, treat, and prevent diseases. With limited resources, debates on where to begin are inevitable. However, such debates rest on a false dichotomy, i.e., that by starting with rare diseases we have forsaken our obligation to address common diseases. To the contrary, what we build and what we learn by implementing PM for rare diseases is extensible to common diseases, not only the immediate goal of better diagnoses but also the long-term challenge of identifying drug targets for common diseases.

**Table 1:** Precision medicine initiatives.

Country	Project/program name	Expected size	Common diseases	Rare diseases (and cancers)
AUSTRALIA	Genomics Health Futures Mission^i^	200,000		✔
CANADA	Canadian Genomics Partnership for Rare Diseases and Canadian Longitudinal Study on Aging^ii^	Nationwide	✔	✔
CHINA	Precision Medicine Initiative^iii^	100,000–100 million	✔	✔
DENMARK	Danish National Genome Center^iv^	60,000	✔	✔
DUBAI	Dubai Genomics^v^	Nationwide	✔	
ESTONIA	Personalised Medicine Programme^vi^	150,000	✔	
EUROPEAN UNION	1+ Million Genomes Initiative^vii^	1,000,000+	✔	
FINLAND	FinnGen^viii^	500,000	✔	
FRANCE	Genomic Medicine France 2025^ix^	235,000 each year	✔	✔
HONG KONG	Hong Kong Genome Project^x^	50,000		✔
ITALY	SardiNIA Project^xi^	60,000	✔	
JAPAN	GEnome Medical alliance Japan^xii^	Nationwide	✔	✔
SAUDI ARABIA	Saudi Human Genome Program^xiii^	100,000	✔	✔
SINGAPORE (AND INTERNATIONAL)	Genome Asia 100 K^xiv^	100,000	✔	
THAILAND	Genomics Thailand^xv^	50,000	✔	✔
TURKEY	Turkish Genome Project^xvi^	100,000–1,000,000	✔	✔
UNITED KINGDOM	100,000 Genomes Project^xvii^	100,000		✔
UNITED KINGDOM	Accelerating Detection of Disease^xviii^	5,000,000	✔	
UNITED STATES	NHGRI Genomic-Medicine^xix^	Nationwide	✔	✔
UNITED STATES	All of Us Research Program^xx^	1,000,000+	✔	

This is an updated version of a previous summary^28^ restricted to projects with over 20,000 genomes (or nationwide efforts where that threshold will likely be exceeded). Funding is not necessarily secure in all instances; thus expected sizes and medical objectives are subject to change. We indicate if there is a focus on diagnosing rare diseases (and cancers). Otherwise, population studies and infrastructure are merged under the common diseases heading as that is their long-term objective.

^i^https://www.health.gov.au/initiatives-and-programs/genomics-health-futures-mission.

^ii^https://www.genomecanada.ca/sites/default/files/cgp4-rd_mission_statement.pdf and https://www.clsa-elcv.ca.

^iii^https://www.bio-itworld.com/2019/08/12/national-genomic-data-initiatives-worldwide-update.aspx.

^iv^https://eng.ngc.dk/news/2019/december/nnf/.

^v^https://www.dha.gov.ae/en/Pages/DubaiGneomicsAbout.aspx.

^vi^https://www.sm.ee/en/news/genome-project-100000-samples-collected-2019-least-50000-more-people-can-join.

^vii^https://ec.europa.eu/digital-single-market/en/european-1-million-genomes-initiative.

^viii^https://www.finngen.fi/en.

^ix^https://solidarites-sante.gouv.fr/IMG/pdf/genomic_medicine_france_2025.pdf.

^x^https://www.fhb.gov.hk/download/press_and_publications/otherinfo/200300_genomic/SCGM_report_en.pdf.

^xi^https://sardinia.nia.nih.gov/.

^xii^https://www.amed.go.jp/en/aboutus/collaboration/ga4gh_gem_japan.html.

^xiii^https://shgp.kacst.edu.sa/index.en.html.

^xiv^https://genomeasia100k.org/.

^xv^https://www.nature.com/articles/d42473–020–00209–6.

^xvi^https://www.bbmri-eric.eu/news-events/turkish-genome-project-launched/.

^xvii^https://www.genomicsengland.co.uk/about-genomics-england/the-100000-genomes-project/.

^xviii^https://www.ukri.org/innovation/industrial-strategy-challenge-fund/accelerating-detection-of-disease/.

^xix^https://www.genome.gov/about-nhgri/Division-of-Genomic-Medicine.

^xx^https://allofus.nih.gov/about/all-us-research-program-overview.

## Rare diseases for the short term

First, what are rare diseases? In the United States, a rare disease is defined as a condition that affects fewer than 200,000 people, or 1 in 1650 people given a current population size of 330 million. This definition is based on the Orphan Drug Act of 1983. In the European Union, rare is defined as fewer than 1 in 2000 people. Most of these diseases present in children, but some present in adults. Although rare in isolation, they are not rare in aggregate. The oft-cited number is that they affect 7% of the population (see Box 1). Most of these diseases are attributed to a single defective gene, i.e., Mendelian, and the identity of this gene is known for many thousands of diseases. The argument for rare diseases is not just that they are better understood. Health economics are more favorable^4^. Because they are so rare, few physicians are trained to recognize them. Hence, they are poorly diagnosed. Affected individuals often endure years of diagnostic odyssey, which is not only fruitless but more expensive than sequencing their genomes upfront^5,6^. For infants admitted to intensive care within the first 100 days of life, sequencing produced diagnostic yields of 36.7%; and in 52.0% of the diagnosed, medical management was affected^7^. Results improved to 50.8% and 71.9%, respectively, when trio sequencing was conducted. Other studies have given similar results^8^.

**Fig. 1:**
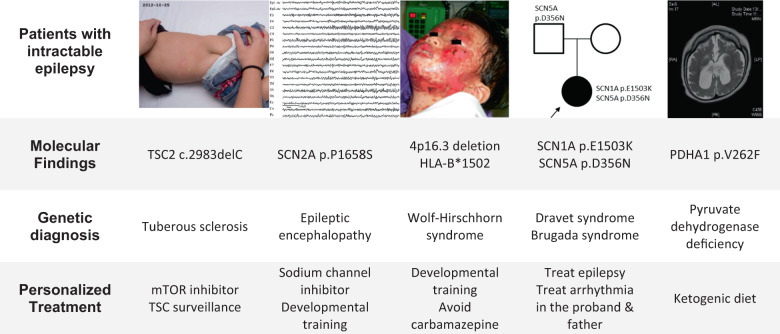
Five different epilepsies, five different treatments. Worldwide, ~50 million people have epilepsy, making it one of the most common neurological diseases. Pediatric‐onset intractable cases are defined by onset before 18-years-of-age with two failed trials of tolerated appropriately-chosen-and-used anti-epileptic drugs (AED) to achieve sustained seizure freedom. An estimated 30% of epilepsy patients fall into this category. Diverse disease etiologies make accurate and specific diagnoses challenging. From the ClinGen Epilepsy Gene Curation Expert Panel^35^, there are 2702 genes associated with epilepsy. A proper molecular diagnosis is therefore essential. Here, we show five examples from the University of Hong Kong (HKU) Paediatric Exome Project, demonstrating how genome medicine enables personalized treatment of difficult epilepsy cases^36^. Informed consent was obtained from the parents for the use of these clinical photographs.

At its heart, PM is about making better diagnoses (see Fig. 1) using the latest technologies to gather more data^9^ and letting that guide our subsequent decisions. To transition from research to routine healthcare requires input from many stakeholders. Every jurisdiction has its own challenges. A good example for how this might be done is the Melbourne Genomics Health Alliance^10^. To diagnose rare diseases, we need sequencing machines, high-throughput computers, and a multi-disciplinary team to manage/interpret the outputs. Most of the costs are in salaries for skilled experts. Much as the invention of magnetic resonance imaging resulted in the creation of specialized referral facilities to acquire and interpret the data, a similar arrangement is used in PM. The referring physician ultimately gets a diagnosis from another physician at the referral facility. Occasionally, the two physicians interact to gather more data before a final diagnosis can be made. Additional experiments are sometimes required to validate novel gene and/or mutation functions, although this is being ameliorated by large-scale phenotyping efforts^11^. The bottleneck, however, is in the training and certification of these multi-disciplinary teams.

**Fig. 2:**
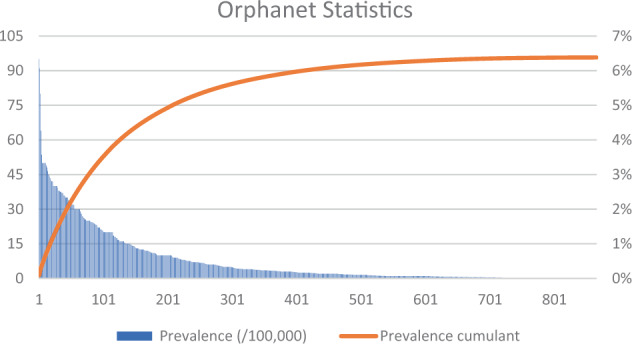
Summary of disease prevalence from Orphanet. Data are sorted from most to least common disease. The solid red line is the cumulant.

To what extent do the lessons of creating such referral facilities for rare diseases transfer to common diseases? Historically, medical progress has often entailed splitting of a disease into a series of sub-diseases, each treated differently. It is not inconceivable that PM will eventually transform any common disease into a series of rare diseases. How we stratify into sub-diseases is still to be determined, and it need not always be genetic, let alone monogenic. PM is simply accelerating this process, with complex data sets that require multi-disciplinary teams to manage and interpret. Hence, the organizational lessons from diagnosing rare diseases are directly transferable to common diseases. Note also that, as we stratify by mutated genes, many therapeutics for otherwise common cancers now qualify for orphan drug status^12^. This was certainly not the intention of the orphan drug laws, and we may need to update these laws. For example, perhaps orphan drug status should be granted based on the number of patients across all indications. The sooner policymakers are warned about this growing issue, the more likely they can deal with the ramifications.

BOX 1: Lower bound for overall prevalence of rare diseases It is very difficult to measure the prevalence for every known rare disease, not only because there are so many, but also, some are so rare we would need to sample a very large population to obtain an accurate estimate. However, it is possible to establish a lower bound for overall prevalence by adding the numbers for all instances where prevalence has been measured. This information is available from the Orphanet website^37^, organized into mutually exclusive categories: worldwide prevalence, worldwide birth prevalence, European prevalence, and European birth prevalence. The sum is 6.38%, and sorted by categories it is 0.78%, 0.81%, 3.54%, and 1.25%, respectively. The oft-cited estimate of 7%, for example in the UK-NHS report “Generation Genome”^38^, comes remarkably close to this bound. However, combining electronic health records with genomics has identified subsets of people with distinct genetic causes for many common diseases, arguing that people with undiagnosed Mendelian diseases are more prevalent than often assumed^39^. One could therefore ask how much larger the true prevalence might be.

We can extrapolate two ways. If the measured prevalences are an unbiased random sampling of rare diseases, and given that there are over 6000 rare diseases, the total would be >50%. We believe this is highly implausible. More likely, the Orphanet website contains the most common diseases. Given how the cumulant is clearly approaching an asymptote after just a few hundred cases (see Fig. 2), the more plausible total is unlikely to be much larger than 7%. An interesting comparison is the fraction of common multifactorial diseases that can be attributed to early-onset familial forms driven by highly penetrant rare variants. A summary of the published estimates, based on extensive genome-wide association studies and reanalyzes of the data, puts this number at about 10%^40^.

## Common diseases in the long term

All that said, the fact remains that other than perhaps cancer, we are not ready to implement PM in routine healthcare for most common diseases. Therein lies the source of the tensions between and within PM initiatives. Ironically, the way out of this conundrum is to redirect the technology created to diagnose rare diseases towards a strategy to find drug targets for common diseases. What we outline here has its roots in a 22-years-old hypothesis on how gene losses can drive evolutionary changes^13^. It is coupled to the realization that human genetics should be a better model of drug action than animal models or cell lines^14^. For previously approved drugs, human genetics is known to be a good predictor of efficacy and adverse effects^15–17^. Given that the mode-of-action with most drugs is to simulate gene loss, this proposal can be encapsulated by the acronym HKMDs, or Human Knockouts as Models of Drug action. PCSK9 inhibitors, first approved in 2015, are the canonical example. They are more effective than statins at lowering serum cholesterol^18^ and were inspired by a discovery that individuals with loss-of-function (LOF) mutations in PCSK9 exhibit low levels of serum LDL and abnormally good cardiovascular health^19^. Other examples are known in coronary artery diseases^20^. In 2019, a drug (romosozumab) that increases bone density was approved for osteoporosis. It was inspired by another rare LOF mutation, in SOST, where the affected individuals have bones so dense they do not break^21,22^. Although we are only aware of a small number of HKMDs (see Table 2), there are reasons to believe they are widespread (see Box 2). Historically, their discovery has been serendipitous, because human geneticists do not as a matter of practice screen for rare phenotypes. People screen themselves and report to a physician if they are sick; but rare individuals with HKMDs are not typically sick, and therefore, have no reason to self-report.

**Table 2:** Examples of known HKMDs with approved drugs.

Gene	LOF	Allele freq.	Effect size	Phenotype	Approved drug	References
APOC3	T	0.0040 (Danish)	44% for TG; 41–36% for CHD	Lower triglyceride and protection from coronary heart disease	(2019) volanesorsen	^29,30^
		0.0067 (European and African)	39% for TG; 40% for CHD			
CCR5	M	0.092 (European)	100% resistant	Resistance to HIV/AIDS	(2007) maraviroc	^31,32^
FAAH	T**	Not available	Not available	Reduced sensitivity to pain and generally lower anxiety levels	Not applicable	^33^
PCSK9	T	0.026 (African)	28% for LDL; 88% for CHD	Lower serum LDL and protection from coronary heart disease	(2015) alirocumab, evolocumab	^18,19^
SOST	M	Not available	Not available	Higher bone density	(2019) romosozumab	^21,22^

The column LOF indicates if the phenotype is observed in heterozygotes (T) or homozygotes (M). For FAAH, the notation T** indicates that the trait requires heterozygous mutations in two different but functionally related loci. FAAH is a promising alternative for pain-relieving drugs inspired by LOFs in SCN9A^34^. Allele frequency and effect size, when provided, come from the cited references.

*TG* triglyceride, *LDL* low-density lipoproteins, *CHD* coronary heart disease.

There are two approaches to make HKMD discovery more systematic^23^. The genotype-first method would sequence a large number of individuals and analyze their genomes for LOFs that might create the opposite of a disease state (e.g., high bone density) or confer protection against disease (e.g., low serum LDL). As electronic health records are finite, recontact permission will be essential to confirm inferred phenotypes. This is being done with consanguineous populations^24^ and at the UK Biobank^25^. The phenotype-first method would ideally screen a much larger population for hypothesized HKMD phenotypes; for example, using social media to entice individuals to self-report. Considering the multifactorial nature of common diseases, we would expect there to be many causes—not all genetic, let alone a LOF—for any given phenotype. Since the people we sequence are not sick, if we cannot identify a promising LOF in one person, we can move on to the next. Once a candidate HKMD is identified, we can use the growing human genome sequencing databases to validate the genotype–phenotype relationship across a larger number of individuals. Importantly, we can ascertain if a certain genetic background (i.e., the population in which the LOF was discovered) is necessary for that phenotype to manifest. This level of validation would be inconceivable with animal models or cell lines.

Of the two approaches, the phenotype-first method is most compatible with PM facilities set up to diagnose rare diseases. Rather than identify rare mutations specific to sick individuals, they would now identify rare mutations specific to individuals with a phenotype that mimics a desired pharmaceutical objective. Anyone with the large-scale capacity to diagnose rare diseases can easily devote 10% of that capacity to screen phenotypically-defined individuals for HKMDs. This flips on its head a prevailing narrative in medical genetics that views LOFs as detrimental to a small number of people. In the future, rare LOFs may be seen as key to drug development that benefits a large number of people.

BOX 2: Human knockouts as models of drug action To argue that HKMDs may be widespread is to dispel three common misperceptions. First, LOF mutations should not be tolerated in evolutionarily conserved genes. Second, the number of naturally occurring LOFs in any particular individual’s genome ought to be small. Third, it is not possible to modify a complex trait in an arbitrary direction simply by inhibiting a gene/protein. Here, we argue that all three propositions are false.

On the first point, systematic deletions of the *Saccharomyces cerevisiae* genome have long established that only one in five yeast genes are necessary for survival^41^. The human version of these experiments was done more recently. Three independent studies on human cell lines demonstrated that only 10% of our 23,425 protein-coding genes are essential for survival^42–44^. Apparently, even for evolutionarily conserved genes, selective pressures to maintain function are weak. On the second point, initial studies on 185 genomes^45^ and 60,706 exomes^46^ estimated that any human individual has 100–85 heterozygous and 20–35 homozygous LOFs, respectively. A more recent analysis of 141,456 genomes and exomes computed the number of individuals needed to find LOFs in every gene^47^. The distribution for heterozygous LOFs peaked at ten thousand individuals, and LOFs were seen in 79.8% of the genes for this particular data set. For homozygous LOFs, the distribution peaked at a hundred million individuals, and even if we sequenced everyone in the world, four thousand genes will have no LOFs. However, drugs rarely (if ever) inhibit their targets completely; hence, the heterozygous distribution may be more appropriate for HKMDs. If so, any city with a million residents will have multiple individuals with LOFs in almost any gene that might ever be targeted for drug development. Notice however that many of these variants will likely be rarer than oft-studied Mendelian alleles.

On the third point, the critical determinant is the extent to which the trait of interest is regulated, with different genes that drive the trait in opposite directions. By analogy, imagine driving with feet simultaneously on the accelerator and brake. To make the car go faster or slower, one can “inhibit” the brake or accelerator, respectively. Most biological processes are indeed regulated; none more so than the complex traits underlying common diseases. To the extent that this is the case, the primary reason why there may not be an HKMD for drug development is the fact that some LOFs are not tolerated, even as heterozygotes. To a first approximation, this is equivalent to saying there is no drug target for the pharmaceuticals industry to inhibit. Other approaches are required (e.g., drugs to simulate gain-of-function).

## Discussion

Some readers will have noticed a contradiction between two of our key points. If a common disease is a series of rare diseases, might that require a series of HKMD-inspired drugs? Much has been written about the genetic and environmental architecture of complex multifactorial diseases^26,27^, and it is dangerous to generalize to all common diseases. However, to the extent that a disease has a common final pathway of phenotypic or clinical expression triggered by many different genetic and environmental factors, one HKMD-inspired drug may be effective for a large fraction of affected individuals. This certainly is the hope for PCSK9 inhibitors, although more years of data are required to see if they improve cardiovascular health under all genetic and environmental backgrounds. The bigger change that we wish to catalyze is the idea that sequencing people without the disease of interest may be a more efficient way to identify drug targets. Finding a LOF that causes a Mendelian disease does not immediately point us towards a drug target, but finding a LOF that confers a pharmaceutically desirable phenotype does. HKMDs need not be inherited. Some might be de novo mutations. Many are likely to be even rarer than the Mendelian disease alleles that have been the focus of so many fruitful studies. The challenge is to define traits worth emulating by drugs, and to phenotypically screen a very large population for people with these traits.

## References

[1] Turro E (2020). Whole-genome sequencing of patients with rare diseases in a national health system. Nature.

[2] Department of Health and Social Care. Matt Hancock announces ambition to map 5 million genomes. (2018).

[3] Shotelersuk V, Tongsima S, Pithukpakorn M, Eu-Ahsunthornwattana J, Mahasirimongkol S (2019). Precision medicine in Thailand. Am. J. Med. Genet. C. Semin. Med. Genet..

[4] Doble B, Schofield DJ, Roscioli T, Mattick JS (2017). Prioritising the application of genomic medicine. NPJ Genom. Med..

[5] Tan TY (2017). Diagnostic impact and cost-effectiveness of whole-exome sequencing for ambulant children with suspected monogenic conditions. JAMA Pediatr..

[6] Farnaes L (2018). Rapid whole-genome sequencing decreases infant morbidity and cost of hospitalization. npj Genomic Med..

[7] Meng L (2017). Use of exome sequencing for infants in intensive care units: ascertainment of severe single-gene disorders and effect on medical management. JAMA Pediatr..

[8] Wright CF, FitzPatrick DR, Firth HV (2018). Paediatric genomics: diagnosing rare disease in children. Nat. Rev. Genet..

[9] Hou Y-CC (2020). Precision medicine integrating whole-genome sequencing, comprehensive metabolomics, and advanced imaging. Proc. Natl Acad. Sci. USA.

[10] Gaff CL (2017). Preparing for genomic medicine: a real world demonstration of health system change. NPJ Genom. Med..

[11] Brommage R, Powell DR, Vogel P (2019). Predicting human disease mutations and identifying drug targets from mouse gene knockout phenotyping campaigns. Dis. Model. Mech..

[12] Miller KL, Lanthier M (2018). Investigating the landscape of US orphan product approvals. Orphanet J. Rare Dis..

[13] Olson MV (1999). When less is more: gene loss as an engine of evolutionary change. Am. J. Hum. Genet..

[14] Plenge RM, Scolnick EM, Altshuler D (2013). Validating therapeutic targets through human genetics. Nat. Rev. Drug Discov..

[15] Nelson MR (2015). The support of human genetic evidence for approved drug indications. Nat. Genet..

[16] King EA, Wade Davis J, Degner JF (2019). Are drug targets with genetic support twice as likely to be approved? Revised estimates of the impact of genetic support for drug mechanisms on the probability of drug approval. PLoS Genet..

[17] Nguyen PA, Born DA, Deaton AM, Nioi P, Ward LD (2019). Phenotypes associated with genes encoding drug targets are predictive of clinical trial side effects. Nat. Commun..

[18] Chaudhary R, Garg J, Shah N, Sumner A (2017). PCSK9 inhibitors: a new era of lipid lowering therapy. World J. Cardiol..

[19] Cohen JC, Boerwinkle E, Mosley TH, Hobbs HH (2006). Sequence variations in PCSK9, low LDL, and protection against coronary heart disease. N. Engl. J. Med..

[20] Musunuru K, Kathiresan S (2019). Genetics of common, complex coronary artery disease. Cell.

[21] Balemans W (2001). Increased bone density in sclerosteosis is due to the deficiency of a novel secreted protein (SOST). Hum. Mol. Genet..

[22] Brunkow ME (2001). Bone dysplasia sclerosteosis results from loss of the SOST gene product, a novel cystine knot-containing protein. Am. J. Hum. Genet..

[23] Narasimhan VM, Xue Y, Tyler-Smith C (2016). Human knockout carriers: dead, diseased, healthy, or improved?. Trends Mol. Med..

[24] Saleheen D (2017). Human knockouts and phenotypic analysis in a cohort with a high rate of consanguinity. Nature.

[25] Emdin CA (2018). Analysis of predicted loss-of-function variants in UK Biobank identifies variants protective for disease. Nat. Commun..

[26] Timpson NJ, Greenwood CMT, Soranzo N, Lawson DJ, Richards JB (2018). Genetic architecture: the shape of the genetic contribution to human traits and disease. Nat. Rev. Genet..

[27] Crouch DJM, Bodmer WF (2020). Polygenic inheritance, GWAS, polygenic risk scores, and the search for functional variants. Proc. Natl Acad. Sci. USA.

[28] Stark Z (2019). Integrating genomics into healthcare: a global responsibility. Am. J. Hum. Genet..

[29] Jørgensen AB, Frikke-Schmidt R, Nordestgaard BG, Tybjærg-Hansen A (2014). Loss-of-function mutations in APOC3 and risk of ischemic vascular disease. N. Engl. J. Med..

[30] TG and HDL Working Group of the Exome Sequencing Project, National Heart, Lung, and Blood Institute et al. Loss-of-function mutations in APOC3, triglycerides, and coronary disease. *N. Engl. J. Med*. **371**, 22–31 (2014). 10.1056/NEJMoa1307095 PMC4180269 24941081

[31] Hütter G (2009). Long-term control of HIV by CCR5 Delta32/Delta32 stem-cell transplantation. N. Engl. J. Med..

[32] Samson M (1996). Resistance to HIV-1 infection in caucasian individuals bearing mutant alleles of the CCR-5 chemokine receptor gene. Nature.

[33] Habib AM (2019). Microdeletion in a FAAH pseudogene identified in a patient with high anandamide concentrations and pain insensitivity. Br. J. Anaesth..

[34] Kingwell, K. Nav1.7 withholds its pain potential. *Nat. Rev. Drug Discov*. (2019) 10.1038/d41573-019-00065-0. 10.1038/d41573-019-00065-0 31048807

[35] Helbig I (2018). The ClinGen Epilepsy Gene Curation Expert Panel—bridging the divide between clinical domain knowledge and formal gene curation criteria. Hum. Mutat..

[36] Tsang MH-Y (2019). Exome sequencing identifies molecular diagnosis in children with drug-resistant epilepsy. Epilepsia Open.

[37] Orphanet Report Series. Prevalence and incidence of rare diseases: diseases listed by decreasing prevalence, incidence, or number of published cases. http://www.orpha.net/orphacom/cahiers/docs/GB/Prevalence_of_rare_diseases_by_decreasing_prevalence_or_cases.pdf (2019).

[38] Davies, S. C. *Annual Report of the Chief Medical Officer 2016: Generation Genome*. https://www.gov.uk/government/publications/chief-medical-officer-annual-report-2016-generation-genome (2017).

[39] Bastarache L (2018). Phenotype risk scores identify patients with unrecognized Mendelian disease patterns. Science.

[40] Torkamani A, Wineinger NE, Topol EJ (2018). The personal and clinical utility of polygenic risk scores. Nat. Rev. Genet..

[41] Giaever G (2002). Functional profiling of the Saccharomyces cerevisiae genome. Nature.

[42] Wang T (2015). Identification and characterization of essential genes in the human genome. Science.

[43] Blomen VA (2015). Gene essentiality and synthetic lethality in haploid human cells. Science.

[44] Hart T (2015). High-resolution CRISPR screens reveal fitness genes and genotype-specific cancer liabilities. Cell.

[45] MacArthur DG (2012). A systematic survey of loss-of-function variants in human protein-coding genes. Science.

[46] Lek M (2016). Analysis of protein-coding genetic variation in 60,706 humans. Nature.

[47] Minikel EV (2020). Evaluating drug targets through human loss-of-function genetic variation. Nature.

